# The SurCOP Procedure for Ventricular Septal Rupture: Analysis of Outcomes and Preoperative Risk Factors to Guide Surgical Timing

**DOI:** 10.1093/icvts/ivag129

**Published:** 2026-04-30

**Authors:** Chunxu Song, Nan Zhang, Jingtao Cao, Jiayi Guo, Jiayi Shi, Kaiwen Xiao, Chao Liu

**Affiliations:** Department of Cardiovascular Surgery, The First Affiliated Hospital of Zhengzhou University, Zhengzhou 450000, China; Department of Cardiovascular Surgery, The First Affiliated Hospital of Zhengzhou University, Zhengzhou 450000, China; Department of Cardiovascular Surgery, The First Affiliated Hospital of Zhengzhou University, Zhengzhou 450000, China; Department of Cardiovascular Surgery, The First Affiliated Hospital of Zhengzhou University, Zhengzhou 450000, China; Department of Cardiovascular Surgery, The First Affiliated Hospital of Zhengzhou University, Zhengzhou 450000, China; Department of Cardiovascular Surgery, The First Affiliated Hospital of Zhengzhou University, Zhengzhou 450000, China

**Keywords:** ventricular septal rupture, acute myocardial infarction, SurCOP procedure, mortality, AMI-to-surgery time

## Abstract

**Objectives:**

The aim of this study was to explore the curative effect of Surgical repair Combining an Occluder and a Patch (SurCOP) procedure on postinfarction ventricular septal rupture and the related preoperative factors of death risk to assist clinical decision-making.

**Methods:**

This retrospective multicentre study included patients with postinfarction ventricular septal rupture who underwent Surgical repair Combining an Occluder and a Patch (SurCOP) procedure from 2017 to 2025. Preoperative variables were analysed using univariable and multivariable logistic regression to identify independent risk factors for 30-day mortality.

**Results:**

A total of 60 patients were included. The 30-day mortality rate was 23.3%. Multivariable analysis identified older age (OR = 1.100, *P* = .032), higher preoperative creatinine (OR = 1.017, *P* = .017), and shorter time from acute myocardial infarction to surgery (OR = 0.894, *P* = .032) as independent risk factors. Mortality was highest (66.7%) when surgery occurred within 7 days of acute myocardial infarction, compared to 19.2% (7-14 days) and 17.8% (>14 days).

**Conclusions:**

The Surgical repair Combining an Occluder and a Patch procedure, may enable earlier surgical intervention in patients with ventricular septal rupture. Given that advanced age, high preoperative creatinine, and a shorter acute myocardial infarction to surgery time are independent risk factors for early death, delaying surgery until around 1-week post-acute myocardial infarction—when haemodynamically tolerated—is a strategy that may improve outcomes.

## Graphical abstract

**Figure:**
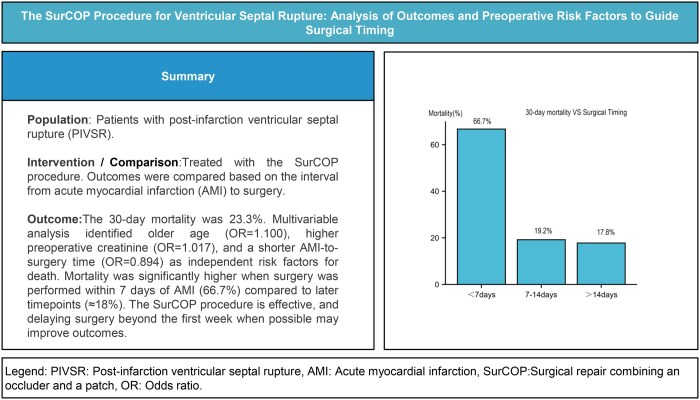

## INTRODUCTION

Ventricular septal rupture (VSR) is a rare and fatal complication following acute myocardial infarction (AMI), and serves as one of the most challenging clinical problems.^1^ Although advances in reperfusion therapy have reduced the incidence of post-infarction ventricular septal rupture (PIVSR) to approximately 0.3%,^2–4^ the mortality rate for patients who develop this complication remains high. It exceeds 90% in those managed conservatively with medical therapy alone and has remained largely unchanged over the past decades.^5^ Percutaneous transcatheter closure can serve as an alternative to surgical repair of VSR. But it is primarily limited to a specific subset of patients with smaller VSR in the subacute or chronic phase.^6^ Traditional surgical repair with a pericardial patch is an effective treatment for VSR; however, it carries a high perioperative mortality. The overall in-hospital surgical mortality rate is approximately 40%.^4^^,^^7^ Our team previously reported a modified surgical technique for managing VSR, which involves the combined use of a closure device and a surgical patch (the SurCOP procedure).^8^ The SurCOP procedure was developed to address the high risk of failure associated with traditional suture repair on friable acute-phase infarcted myocardium. It utilizes an occluder as an anchoring structure to reduce tissue stress, while the occluder’s rigid frame ensures stable patch fixation. Deployment under direct vision allows for precise positioning and, when indicated, facilitates concomitant cardiac procedures. We conducted a retrospective analysis of the preoperative clinical data from VSR patients who underwent the SurCOP procedure. To identify preoperative risk factors associated with prognosis and mortality in VSR patients treated with the SurCOP procedure, in order to provide insights for perioperative management in patients with VSR.

## METHODS

### Data collection and ethics statement

A retrospective study was conducted on 60 patients with VSR who were consecutively managed at the participating centres by our surgical team and all of whom underwent the SurCOP procedure. The clinical preoperative data were collected from patients admitted to the First Affiliated Hospital of Zhengzhou University (*n* = 55), Wuhan Asia Heart Hospital (*n* = 3), and a hospital in the Gansu region (*n* = 2) between January 2017 and August 2025. To provide a comparative reference, we retrospectively collected clinical data from PIVSR patients who underwent conventional surgical repair (David procedure) at our centre between May 2012 and June 2025 (*n* = 24). Their baseline characteristics and surgical outcomes are presented in Table S1. Intraoperative and postoperative data, including cardiopulmonary bypass time, aortic cross-clamp time, and postoperative complications, are provided in Table S2. The study protocol was approved by the First Affiliated Hospital of Zhengzhou University Ethics Committee (Approval No. SS-2019–001). Given the retrospective nature of this study, the ethics review board has waived the requirement for obtaining written informed consent documents.

### Screening criteria

The patient inclusion criteria were as follows: (1) Confirmed diagnosis of AMI, defined as electrocardiographic findings (ST-segment elevation ≥0.1 mV in 2 contiguous limb leads or >0.2 mV in 2 contiguous precordial leads) along with elevated cardiac biomarkers. (2) Echocardiography demonstrating a discontinuity or loss of echo continuity in the interventricular septum, accompanied by a left-to-right shunt. (3) VSR patients treated with the SurCOP procedure. The patient exclusion criteria were as follows: (1) Concomitant presence of other mechanical complications, such as ventricular free wall rupture or chordae tendineae rupture. (2) Previous history of percutaneous VSR closure. (3) History of isolated surgical repair with a biological patch. (4) Acute phase of a cerebrovascular accident. (5) Congenital heart disease with a ventricular septal defect (VSD).

### Surgical technique

**Figure 1.**
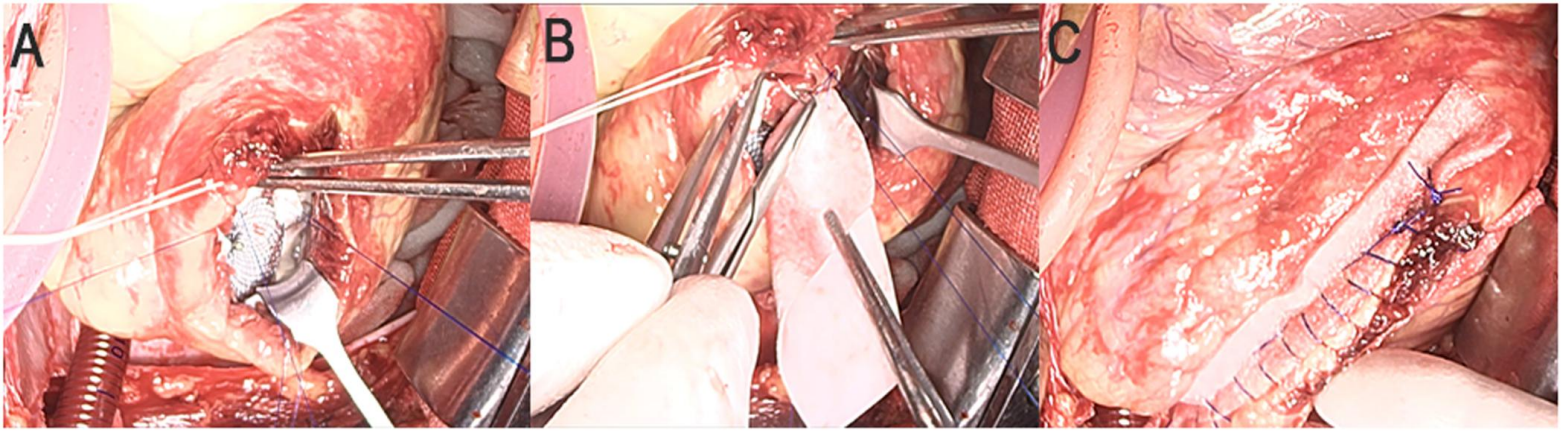
Intraoperative Image of the SurCOP Procedure. (A) Deployment of the PDA occluder across the VSD, secured with interrupted pledgetted sutures at the 12, 3, 6, and 9 o'clock positions. (B) Placement of a pericardial patch on the left ventricular side, anchored with a continuous 4–0 Prolene suture. (C) Ventriculotomy closure using the sandwich technique with felt strips, performed concomitantly in patients with a ventricular aneurysm.

Under general anaesthesia, cardiopulmonary bypass was established through a median sternotomy with cannulation of the ascending aorta and both the superior and inferior venae cavae. A left heart vent was placed via the right superior pulmonary vein. Following cross-clamping of the ascending aorta, a cardioplegic solution was administered to induce cardiac arrest. Preoperative coronary angiography (CAG) was performed to assess the severity of stenosis in the culprit vessel. The findings guided the decision on whether to perform concomitant coronary artery bypass grafting (CABG). If severe regurgitation of the mitral or tricuspid valve was identified, valve repair or replacement was performed concomitantly. An appropriate patent ductus arteriosus (PDA) occluder was selected based on the size of the VSR. PDA occluder sizing was based on intraoperative measurement of the VSR, with the waist diameter usually within  ± 2 mm of the defect diameter. During deployment, the proximal disc was positioned facing the left ventricular side, and the distal disc faced the right ventricular side, allowing the waist of the appropriately sized occluder to nestle securely within the defect. The occluder was then fixed in place with interrupted pledgetted sutures placed at the 12, 3, 6, and 9 o’clock positions along its rim (Figure 1A). Finally, the pericardial patch was placed on the left ventricular side, approximated to the rim of the occluder, and secured with a continuous 4–0 Prolene suture (Figure 1B).^8^ In patients with a concomitant ventricular aneurysm, aneurysm resection was performed concomitantly, and the ventriculotomy was closed using the sandwich technique with felt strips (Figure 1C). A step-by-step surgical video of the SurCOP procedure is available in the supplementary materials.

### Statistical analysis

**Figure 2.**
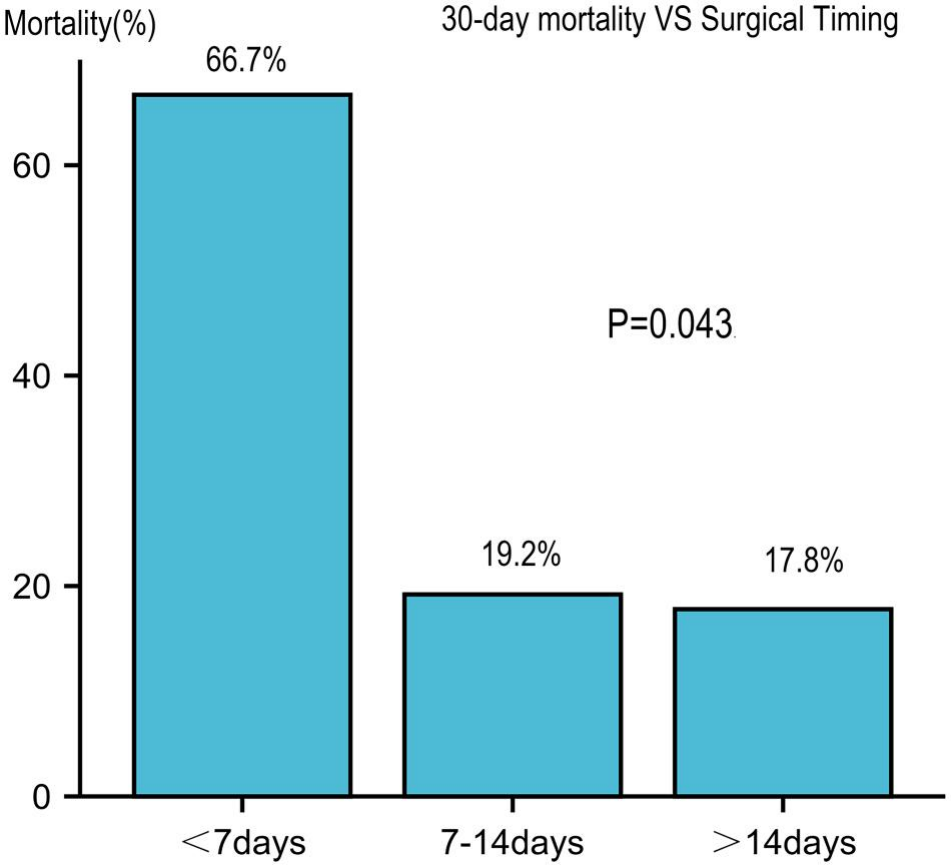
30-Day Postoperative Mortality Stratified by AMI-to-Surgery Time

**Figure 3.**
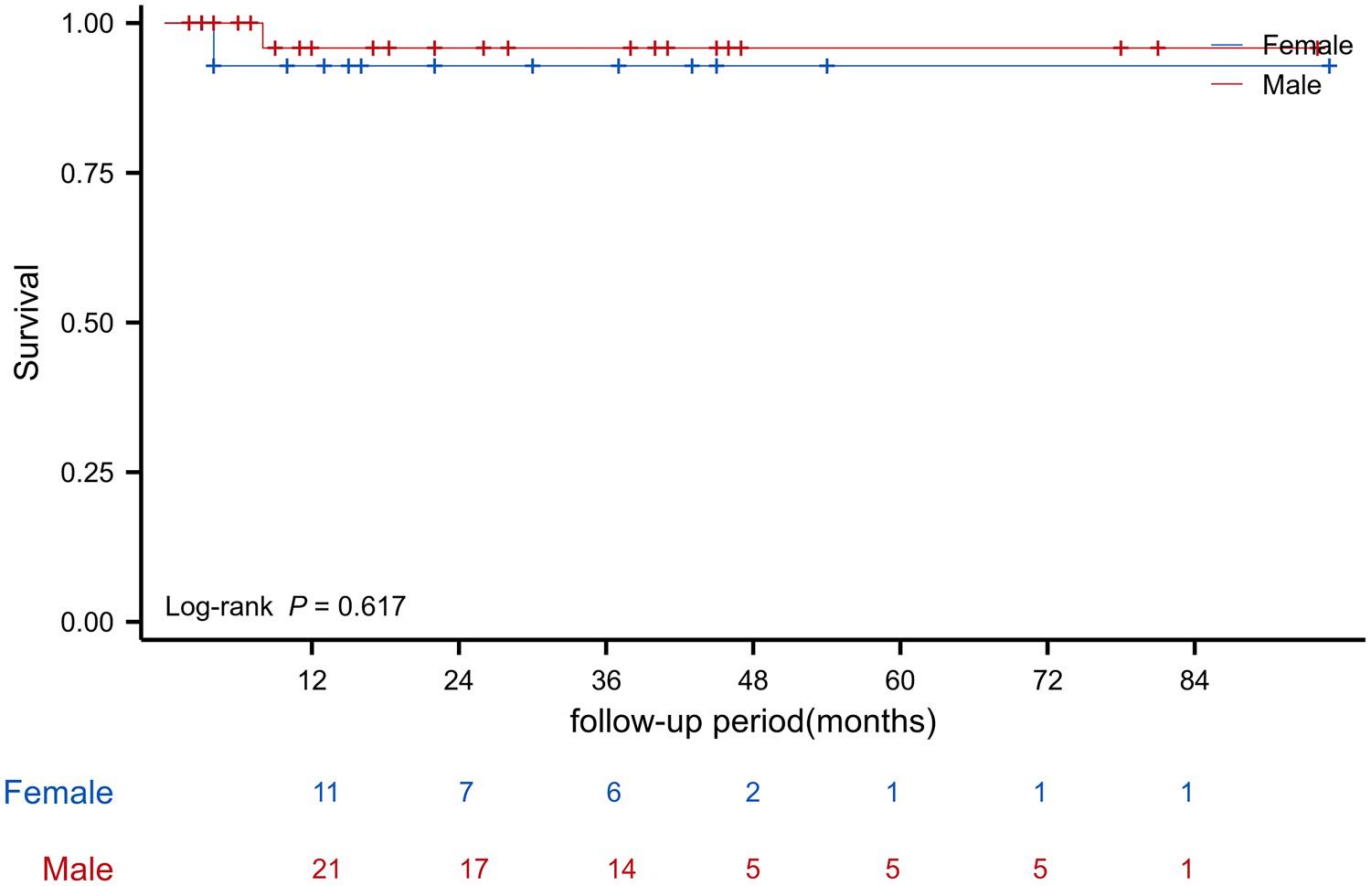
Kaplan-Meier Survival Curves Stratified by Sex after SurCOP Procedure

First, univariable logistic regression analysis is carried out to determine the potential inducing risk factors of VSR. Continuous variables were expressed as mean (standard deviation) or median [interquartile range (IQR)], and categorical variables as frequency (percentage). The results are expressed as odds ratios (OR) along with 95% CI. Variables with *P* value <0.1 in the univariable analysis were included in the multivariable logistic regression model. Two variable selection methods, forward stepwise and backward stepwise, were adopted. The final models obtained by the 2 methods were the same, indicating that the core variables were relatively stable. All the variables in the final model were independent risk factors, and the *P* value < 0. 0.05 was statistically significant. All statistical analyses (stats anal ses) were performed using SPSS 27.0 (IBM, Armonk, New York, USA). The bar chart for 30-day mortality stratified by AMI-to-surgery time (Figure 2) and the Kaplan-Meier survival curves stratified by sex (Figure 3) were generated using R software version 4.2.1.

## RESULTS

### Baseline clinical characteristics

Totally, 60 patients with VSR who underwent the SurCOP procedure were enrolled between January 2017 and August 2025. The mean age was 63.8 ± 9.264 years, with 39 males (65%) and 21 females (35%). The study showed the comorbidities of hypertension in 32 patients (53.3%) and diabetes mellitus in 18 patients (30%). The all-cause 30-day mortality rate was 23.3% (14/60), with 46 patients surviving. Preoperatively, 26 patients (43.3%) had undergone percutaneous coronary intervention (PCI), 23 (38.3%) required intra-aortic balloon pump (IABP) placement, and 4 (6.7%) received extracorporeal membrane oxygenation (ECMO) support. Additionally, 3 patients (5.0%) required continuous renal replacement therapy (CRRT), and 7 (11.7%) had a history of cerebrovascular disease. The mean preoperative left ventricular ejection fraction (LVEF) was 48.97% ± 9.54%. The VSR diameter was 17 mm (IQR: 10, 20 mm), the preoperative serum creatinine level was 91.00 µmol/l (IQR: 77.43, 134.30 µmol/l), and the time interval from AMI onset to surgery was 13 days (IQR: 8, 22 days). Most patients (*n* = 42, 70%) were in Killip class III or IV prior to surgery. The preoperative baseline characteristics of the survivor and non-survivor groups were detailed in Table 1. The results of the univariable logistic regression analysis are presented in Table 2.

**Table 1.** Baseline Characteristics of Patients with VSR

Parameter	Overall (*n* = 60)	Survivors (*n* = 46)	Non-survivors (*n* = 14)
Male, *n* (%)	39 (65)	30 (65.2)	9( 64.3)
Age, years	63.80 ± 9.26	62.46 ± 9.76	68.21 ± 5.70
Smoking, *n* (%)	21 (35)	18 (39.1)	3 (21.4)
Alcohol use, *n* (%)	12 (20)	11 (23.9)	1 (7.1)
Admission examination			
Systolic blood pressure, mmHg	107.19 ± 15.03	106.11 ± 14.23	110.92 ± 17.63
Diastolic blood pressure, mmHg	67.5 (61.5,76)	67 (61,78)	68.5 (65,76)
Heart rate, beats/min	97.50 ± 19.51	97.02 ± 20.68	99.07 ± 15.61
Past medical history			
Hypertension, *n* (%)	32 (53.3)	24 (52.2)	8 (57.1)
Diabetes mellitus, *n* (%)	18 (30)	13 (28.3)	5 (35.7)
Cerebrovascular accident, *n* (%)	7 (11.7)	3 (6.5)	4 (28.6)
Killip class			
I	1 (1.7)	1 (2.2)	0 (0)
II	17 (28.3)	15 (32.6)	2 (14.3)
III	20 (33.3)	15 (32.6)	5 (35.7)
IV	22 (36.7)	15 (32.6)	7 (50)
Preoperative status			
Preoperative shock, *n* (%)	12 (20)	7 (15.2)	5 (35.7)
Previous PCI, *n* (%)	26 (43.3)	22 (47.8)	4 (28.6)
Preoperative IABP, *n* (%)	23 (38.3)	14 (30.4)	9 (64.3)
Preoperative CRRT, *n* (%)	3 (5)	1 (2.2)	2 (14.3)
Preoperative ECMO, *n* (%)	4 (6.7)	2 (4.3)	2 (14.3)
Preoperative laboratory tests			
Total cholesterol, mmol/l	3.68 ± 0.85	3.70 ± 0.85	3.62 ± 0.88
Triglycerides, mmol/l	1.12 (1.02,1.68)	1.22 (1.06,1.67)	1.145 (0.99,1.60)
HDL-C, mmol/l	0.875 (0.678,1.14)	0.885 (0.68,1.17)	0.735 (0.665,0.99)
LDL-C, mmol/l	2.32 ± 0.83	2.33 ± 0.83	2.30 ± 0.84
Preoperative creatinine, μmol/l	91.0 (77.4,134.3)	87.5 (77.0,120.0)	143.5 (107,180)
BNP, ng/l	8370 (4019.50,15218.0)	7132.85 (2875.00,13095.00)	12 862 (6721,19414)
Lactate (latest pre-op), mmol/l	1.50 (1.18,2.36)	1.50 (1.20,2.20)	1.70 (1.10,4.90)
Cardiac structure & function			
Left ventricular ejection fraction,%	48.97 ± 9.54	48.57 ± 9.30	50.39 ± 10.63
VSR diameter, mm	17 (10,20)	18 (10,20)	15 (12,19)
Number of diseased coronary arteries			
Single-vessel disease *n* (%)	41 (68.3)	33 (71.7)	8 (57.1)
Two-vessel disease *n* (%)	9 (15)	5 (10.9)	4 (28.6)
Three-vessel disease *n* (%)	10 (16.7)	8 (17.4)	2 (14.3)
VSR-to-surgery time, days	9 (5,15)	6 (9.5,15)	4.5 (2,15)
AMI-to-surgery time, days	13 (8,22)	14 (9,24)	10 (5,16)

**Table 2.** Univariable Logistic Regression Analysis Analysis of Factors Associated With 30-Day Mortality

Parameter	OR (95% CI)	*P-*value
Male, *n* (%)	1.04 (0.30-3.64)	.95
Age, years	1.08 (1.00-1.17)	**.048**
Smoking, *n* (%)	0.42 (0.10-1.73)	.23
Alcohol use, *n* (%)	0.25 (0.03-2.09)	.2
Admission examination		
Systolic blood pressure, mmHg	1.02 (0.98-1.07)	.31
Diastolic blood pressure, mmHg	0.99 (0.94-1.04)	.6
Heart rate, beats/min	1.01 (0.98-1.04)	.73
Past medical history		
Hypertension, *n* (%)	1.22 (0.37-4.08)	.74
Diabetes mellitus, *n* (%)	1.41 (0.40-5.01)	.6
Cerebrovascular accident *n* (%)	5.73 (1.10-29.78)	**.038**
Killip class	1.83 (0.84-3.99)	.128
I		
II		
III		
IV		
Preoperative status		
Preoperative Shock, *n* (%)	3.10 (0.80-12.03)	.103
Previous PCI, *n* (%)	0.44 (0.12-1.59)	.21
Preoperative IABP, *n* (%)	4.11 (1.17-14.52)	**.028**
Preoperative CRRT, *n* (%)	7.50 (0.63-89.87)	.112
Preoperative ECMO, *n* (%)	3.67 (0.47-28.81)	.22
Preoperative laboratory tests		
Total cholesterol, mmol/l	0.89 (0.41-1.93)	.77
Triglycerides, mmol/l	0.87 (0.25-3.16)	.85
HDL-C, mmol/l	0.34 (0.03-3.72)	.38
LDL-C, mmol/l	0.96 (0.44-2.11)	.92
Preoperative creatinine, μmol/l	1.01 (1.00-1.02)	**.017**
BNP, ng/l	1.00 (0.998-1.001)	.25
Lactate (latest pre-op), mmol/l	1.19 (0.96-1.48)	.113
Cardiac structure & function		
Left ventricular ejection fraction, %	1.02 (0.96-1.09)	.54
VSR diameter, mm	0.98 (0.88-1.09)	.7
Number of diseased coronary arteries	1.21 (0.57-2.57)	.62
Single-vessel disease, *n* (%)		
Two-vessel disease, *n* (%)		
Three-vessel disease, *n* (%)		
VSR-to-surgery time, days	0.94 (0.86-1.03)	.157
AMI-to-surgery time, days	0.93 (0.86-1.01)	**.074**

Abbreviations: CRRT, continuous renal replacement therapy; ECMO, extracorporeal membrane oxygenation; HDL, high-density lipoprotein; IABP, intra-aortic balloon pump; LDL, low-density lipoprotein; NT-proBNP, N-terminal pro-B-type natriuretic peptide; LVEF, left ventricular ejection fraction; PCI, percutaneous coronary intervention.

### Univariable and multivariable logistic regression analyses

The univariable analysis initially identified 6 variables associated with 30-day postoperative mortality (*P* < 0.1), including: age, history of cerebrovascular accident, preoperative use of IABP, preoperative creatinine level, and time from AMI to surgery. All these variables were shown in the multivariable logistic regression model for further selection. The results demonstrated that time from AMI to surgery, age, and preoperative creatinine level functioned as independent risk factors for mortality following the SurCOP procedure (all *P* < .05), as detailed in Table 3.

**Table 3.** Multivariable Analysis of Preoperative Parameters for Predicting 30-Day Postoperative Mortality

Parameter	β	SE	Wald	OR	95% CI	*P* -value
AMI-to-surgery time, days	–0.112	0.053	4.579	0.894	0.806-0.991	.032
Age, years	0.096	0.045	4.597	1.1	1.008-1.201	.032
Preoperative creatinine, μmol/l	0.016	0.007	5.687	1.017	1.003-1.030	.017
Intercept	–7.842	3.202	5.999	0		.014

### Comparison with historical control group

To provide a reference for outcomes, we retrospectively identified 24 patients who underwent conventional surgical repair (David procedure) for PIVSR at our centre during the same study period and included them as a historical control group. Baseline characteristics of the SurCOP and control groups are summarized and compared in Table S1. Compared with the SurCOP cohort, patients in the control group had a longer median time from AMI to surgery [41.5 days (IQR: 26.5-75) vs 13 days (IQR: 8-22)], a lower rate of preoperative IABP use (8.3% vs 38.3%), and higher proportions of diabetes mellitus (50% vs 30%) and preoperative shock (33.3% vs 20%). Other baseline variables, including age, sex, Killip class, LVEF, and VSR diameter, were generally comparable between the two groups. The 30-day all-cause mortality rate was 23.3% (14/60) in the SurCOP group and 25.0% (6/24) in the control group.

### Association of surgical timing with mortality

To further elucidate the impact of surgical timing, patients were stratified into three groups based on the interval from AMI to surgery: <7 days, 7-14 days, and >14 days. Group comparisons were done via the Chi-square or Fisher’s exact test. A significant difference in mortality was discovered among the groups (*P* = .043). Specifically, as illustrated in Figure 2, the mortality rate was substantially higher in the <7 days group (66.7%, 4/6) compared to the 7-14 days group (19.2%, 5/26) and the >14 days group (17.8%, 5/28). The mortality rates between the 7-14 days and >14 days groups were comparable as detailed in Table 4.

**Table 4.** Patient 30-Day Postoperative Survival Stratified by AMI-to-Surgery Time

	AMI-to-surgery time, days			Overall
	< 7	7-14	>14	
30-day postoperative				
Survivors	1	22	23	46
Non-survivors	4	5	5	14
Overall	5	27	28	60

### Long-term follow-up and survival

As of October 2025, follow-up was completed for all 46 patients who survived the initial 30-day postoperative period, with a median follow-up duration of 26 months (IQR: 8.75-45.25 months) and no patients lost to follow-up. During the follow-up period, only 2 deaths occurred: 1 male patient died 8 months after surgery due to small cell lung cancer, and 1 female patient died 4 months after surgery from an unknown cause. The remaining 44 patients were alive at the end of follow-up. No device-related complications, including occluder infection, thrombosis, or migration, were observed, and no patient required reoperation. The Kaplan-Meier survival curve for the entire cohort is shown in Figure 3.

## DISCUSSION

PIVSR is a relatively rare but serious complication. The occurrence of PIVSR has been reduced due to the progress of myocardial reperfusion therapy and interventional techniques, but the mortality rate is still relatively high. Surgical intervention is still the main treatment method, especially for patients with haemodynamic instability.^1–3^ This study retrospectively analyzed 60 VSR patients who underwent the SurCOP procedure to find the key preoperative risk factors affecting the 30-day mortality after surgery. It was found that the all-cause 30-day mortality rate was 23%, which is lower than the mortality rates reported for conventional surgical repair (eg, the 44.2% reported by Giblett et al.).^9^ This advantage may be partially attributable to the earlier surgical intervention. This tension-reducing advantage may explain the earlier surgery in the SurCOP cohort. As the median time from acute myocardial infarction to surgery in our cohort was shorter than that in the conventional surgery control group at our centre [13 days (IQR: 8-22) vs 41.5 days (IQR: 26.5-75)], suggesting a potential benefit of the SurCOP procedure in the management of VSR.A staged approach—initial percutaneous closure (even if incomplete) to reduce shunt and stabilize the patient, followed by delayed surgical repair—has been reported.^10^ While this avoids acute-phase surgery, it requires 2 procedures and carries risks of device embolization or residual shunt. The SurCOP procedure offers single-stage definitive repair under direct vision, enabling precise positioning, concomitant procedures, and immediate confirmation of repair integrity. In our cohort, 5 patients had residual shunts (all <3 mm), which were haemodynamically insignificant. Additionally, the SurCOP procedure allows for simultaneous concomitant cardiac procedures when indicated. Treatment selection should be individualized based on haemodynamic status, VSR anatomy, and institutional expertise.

In the research, univariable and multivariable logistic regression analyses showed that age, preoperative creatinine level, and the time from AMI to surgery were independent risk factors for 30-day postoperative mortality. The preoperative condition of VSR patients can affect the clinical outcome. An analysis of the STS database of 2876 VSR patients showed that age, preoperative dialysis, preoperative IABP support, and the time interval from AMI to surgical repair (≥21 days vs shorter) were independent risk factors for 30-day all-cause mortality after surgery.^7^

The 2013 AHA and 2017 ESC guidelines for myocardial infarction both recommend proactive preoperative use of intra-aortic balloon pump (IABP) in patients with VSR.^11^^,^^12^ In our study, preoperative IABP was employed in 23 patients (38.3%). Its use was more frequent in the non-survivor group (9/14, 64.3%) compared to the survivor group (14/46, 30.4%). In univariable analysis, the use of IABP is a risk factor, but this is not the case in multivariable models. This may mean that it reflects the severity of preoperative haemodynamic instability rather than being an independent risk factor.

Age in this study was an independent risk factor. The data showed that the average age of non-survivors was higher than that of survivors (68.21 years vs 62.46 years, *P* = .009). This study confirmed by multivariable logistic regression that for every 1-year increase in age, the mortality rate within 30 days after surgery increases by 10% (OR = 1.100). Advanced age is a strong predictor of adverse outcomes related to SurCOP procedure. The negative impact of age on postoperative prognosis is explainable because it originates from the general reduction in the physiological reserve of key organ systems (such as the heart, lungs, and kidneys) related to age.^13^ Elderly patients have various coexisting conditions, such as poor multi-organ function and fragile blood vessels. These conditions make them more difficult to withstand major physiological stress, such as surgical trauma, anaesthesia, and extracorporeal circulation.^14^ These mechanisms increase the perioperative risks of elderly patients. The decrease in physiological reserve makes them prone to postoperative functional decline, which may cause or aggravate serious complications, such as heart failure, acute kidney injury (AKI), respiratory failure.^13^^,^^14^ Although the SurCOP procedure may be able to reduce surgical risks. Elderly patients are still vulnerable groups and need special care during the perioperative period; more comprehensive preoperative evaluation and optimization are needed.

VSR leads to left-to-right intracardiac shunting and haemodynamic instability. This state may cause insufficient circulating blood volume and may also lead to insufficient renal perfusion, thereby increasing serum creatinine levels.^15^^,^^16^ Preoperative elevation of creatinine indicates poor previous renal function in patients. Studies have shown that in cardiac surgery, preoperative elevation of serum creatinine increases the risk of postoperative AKI.^17^ The median preoperative creatinine of non-survivors is much higher than that of survivors (143.5 μmol/l vs 87.5 μmol/l), and the *P* value is .040. In the multivariable analysis, the adjusted OR of preoperative creatinine is 1.017 (*P* = .017). Studies have shown that Renal insufficiency can affect drug metabolism, fluid management and regulation of inflammatory response, which increases the risk of postoperative AKI and multiple organ dysfunction syndrome (MODS).^18^^,^^19^

The 2023 European Guidelines for the Management of Acute Coronary Syndromes proposed that surgery should be performed immediately for patients with refractory shock or persistent right ventricular dysfunction. For other patients, surgery should be delayed if conditions permit, ideally after the seventh day after VSR diagnosis.^20^ Many studies have shown that the mortality rate of VSR repair surgery performed within 7 days is 40%-90%, while the mortality rate drops to 10%-40% when performed after 7 days.^10^^,^^21–23^ Some studies (there may be selection and follow-up biases) have obtained biased results, and high-risk patients often die before surgery. Delayed closure is to allow the infarct area to form a more solid scar to stabilize the sutmure.^9^^,^^24^ Multivariable analysis identified AMI-to-surgery time as an independent protective factor for 30-day mortality (OR = 0.894 per day, *P* = .032), indicating that longer delay from AMI to surgery is associated with lower mortality. Stratified analysis confirmed this trend: mortality was 66.7% in patients operated within 7 days, compared to 19.2% at 7-14 days and 17.8% after 14 days (*P* = .043).^20^

VSR typically occurs within 48 hours of AMI,^24^^,^^25^ creating a clinical dilemma: while delaying surgery allows tissue healing, patients may deteriorate during the waiting period. In this context, the SurCOP procedure may offer a technical advantage. By using an occluder as an anchoring structure, it reduces tension on friable infarcted myocardium, potentially enabling safer repair in the subacute phase (7-14 days post-AMI). This allows surgery earlier than the traditional 3-4 week window without excessively increasing operative risk. Thus, for haemodynamically stable patients, delaying surgery until at least 7 days post-AMI—but not necessarily several weeks—may represent an optimal balance.

## LIMITATIONS

This study found that there was no significant association between postoperative mortality and preoperative factors such as LVEF, VSR size, and the number of diseased coronary arteries. This absence may be due to a small sample size or bias in patient selection. This study represents the experience of a single team and technique, and it has limitations such as retrospective design, small sample size, and multicentre origin, which may lead to selection bias. In addition, confounding factors such as infarct size and medications were not included in the analysis, which may affect the comprehensiveness of the results. Additionally, echocardiographic data were incomplete for some patients, highlighting the need for standardized echocardiographic follow-up protocols in future prospective studies. Future studies need larger-scale prospective studies, and PIVSR animal models are warranted to further validate these conclusions.

## CONCLUSION

After review and analysis, it is found that using the SurCOP procedure may enable earlier surgical intervention in patients with VSR. The results also point out that advanced age, high creatinine value, and interval from AMI to surgery are independent risk factors for all-cause death within 30 days after surgery.

The research results show that for VSR patients whose haemodynamics can be maintained, it is necessary to actively optimize renal function and maintain the stability of the whole body, and delay surgical intervention to about 1 to 2 weeks after AMI. This may be able to improve the surgical efficiency and the prognosis of patients. SurCOP procedure is a feasible option for high-risk patients, but its long-term effect needs to be re-verified by larger-scale prospective studies.

## AUTHORS CONTRIBUTIONS

Chunxu Song (Conceptualization, Data curation, Formal analysis, Writing—original draft, Writing–review & editing), Nan Zhang (Conceptualization, Data curation, Writing—original draft, Visualization), Jingtao Cao (Conceptualization, Methodology, Validation), Jiayi Guo (Investigation, Project administration, Validation), Jiayi Shi (Data curation, Resources), Kaiwen Xiao (Data curation, Methodology), and Chao Liu (Investigation, Methodology, Writing–review & editing)

## Supplementary Material

ivag129_Supplementary_Data

## Data Availability

The data resources adopted and deeply analyzed in the experimental process of this topic can all be obtained by contacting the corresponding author of this article on the premise of meeting the reasonable application conditions.
