# Supplementary material for: The SurCOP Procedure for Ventricular Septal Rupture: Analysis of Outcomes and Preoperative Risk Factors to Guide Surgical Timing
Source: Interdiscip Cardiovasc Thorac Surg. 2026 Apr 30;41(6):ivag129. doi: 10.1093/icvts/ivag129 (PMC13263527; doi:10.1093/icvts/ivag129)
Supplement: ivag129_Supplementary_Data [file ivag129_supplementary_data.zip › Table_S1.docx]

| Parameter | Overall (n=24) | Survivors（n=18） | Non-survivors（n=6） | OR(95% CI) | p-value |
| --- | --- | --- | --- | --- | --- |
| Male, n(%) | 16(66.7) | 12(66.7) | 4(66.7) | 1.0(0.14-7.10) | 1 |
| Age, years | 60.62±8.26 | 61.11±7.99 | 67.17±7.96 | 1.11(0.97-1.28) | 0.131 |
| Smoking, n (%) | 7(29.2) | 4(22.2) | 3(50) | 3.50(0.50-24.56) | 0.21 |
| Alcohol Use, n (%) | 6(25) | 4(22.2) | 2(33.3) | 1.75(0.23-13.31) | 0.59 |
| Admission Examination |  |  |  |  |  |
| Systolic Blood Pressure,  mm Hg | 113.58±14.96 | 114.5±15.14 | 110.83±15.43 | 0.98(0.92-1.05) | 0.6 |
| Diastolic Blood Pressure,  mm Hg | 70.46±9.34 | 74.83±9.41 | 70.33±9.91 | 0.98(0.89-1.09) | 0.73 |
| Heart Rate, beats/min | 92.17±19.21 | 88.28±15.87 | 103.83±24.98 | 1.05(0.99-1.11) | 0.107 |
| Past Medical History |  |  |  |  |  |
| Hypertension, n (%) | 15(62.5) | 12(66.7) | 3(50) | 0.50(0.08-9.27) | 0.47 |
| Diabetes Mellitus, n (%) | 12(50) | 7(38.9) | 5(83.3) | 7.86(0.75-82.13) | 0.085 |
| cerebrovascular accident n  (%) | 1(4.2) | 0(0) | 1(16.7) | - | 1 |
| Killip Class |  |  |  | 4.71(0.88-25.05) | 0.069 |
| I | 1(4.2) | 1(5.6) | 0(0) |  |  |
| II | 5(20.8) | 5(27.8) | 0(0) |  |  |
| III | 12(50) | 9(50) | 3(50) |  |  |
| IV | 6(25) | 3(16.7) | 3(50) |  |  |
| Preoperative Status |  |  |  |  |  |
| Preoperative Shock, n (%) | 9(33.3) | 4(22.2) | 5(83.3) | 17.5(1.56-196.32) | 0.02 |
| Previous PCI, n (%) | 10(41.7) | 9(50) | 1(16.7) | 0.20(0.02-2.07) | 0.177 |
| Preoperative IABP, n (%) | 2(8.3) | 0(0) | 2(33.3) |  |  |
| Preoperative CRRT, n (%) | 0(0) | 0(0) | 0(0) |  |  |
| Preoperative ECMO, n (%) | 0(0) | 0(0) | 0(0) |  |  |
| Preoperative Laboratory  Tests |  |  |  |  |  |
| Preoperative Creatinine, μ mol/L | 96.12±57.93 | 79.52±26.66 | 143.17±97.32 | 1.04(0.99-1.08) | 0.096 |
| Lactate (latest pre-op),  mmol/L | 1.77±1.87 | 1.17±0.41 | 3.50±3.17 | 8.07(0.55-118.67) | 0.128 |
| Cardiac Structure &  Function |  |  |  |  |  |
| Left Ventricular Ejection  Fraction, % | 48.67±11.27 | 47.61±11.87 | 51.83±9.45 | 1.04(0.95-1.13) | 0.42 |
| VSR Diameter, mm | 15.71±6.89 | 14.67±5.38 | 18.83±10.21 | 1.10(0.95-1.27) | 0.21 |
| Number of Diseased  Coronary Arteries |  |  |  | 0.77(0.21-2.76) | 0.687 |
| Single-vessel Disease n  (%) | 13(54.2) | 9(50) | 4(66.7) |  |  |
| Two-vessel Disease n (%) | 6(25) | 5(27.8) | 1(16.7) |  |  |
| Three-vessel Disease n  (%) | 4(16.7) | 3(16.7) | 1(16.7) |  |  |
| VSR-to-Surgery Time, days | 35.20±26.00 | 41.02±26.04 | 17.77±17.84 | 0.94(0.88-1.01) | 0.075 |
| AMI-to-Surgery Time, days | 41.5(26.5,75) | 51.5(39,81) | 24(14,33) | 0.93(0.87-1.00) | 0.058 |
