# Supplementary material for: The SurCOP Procedure for Ventricular Septal Rupture: Analysis of Outcomes and Preoperative Risk Factors to Guide Surgical Timing
Source: Interdiscip Cardiovasc Thorac Surg. 2026 Apr 30;41(6):ivag129. doi: 10.1093/icvts/ivag129 (PMC13263527; doi:10.1093/icvts/ivag129)
Supplement: ivag129_Supplementary_Data [file ivag129_supplementary_data.zip › Table_S2.docx]

|  | Overall (n=60) | Survivors(n=46) | Non-survivors(n=14) | OR (95% CI) | *p-*value |
| --- | --- | --- | --- | --- | --- |
| Intraoperative​ |  |  |  |  |  |
| Concomitant Procedures |  |  |  |  |  |
| Coronary artery bypass grafting  (CABG) | 32(53.3) | 26(56.5) | 6(42.9) | 0.55(0.16-1.84) | 0.33 |
| Mitral valvuloplasty | 3(5) | 2(4.3) | 1(7.1) | 1.69(0.14-20.19) | 0.68 |
| Tricuspid valvuloplasty | 11(18.3) | 8(17.4) | 3(21.4) | 1.30(0.29-5.73) | 0.73 |
| Ventricular aneurysm resection | 54(90) | 43(93.5) | 11(78.6) | 0.26(0.05-1.45) | 0.123 |
| PDA Occluder Waist Diameter, mm | 20(16, 20) | 20(16, 22) | 20(16, 24) | 1.01(0.88-1.15) | 0.94 |
| Cardiopulmonary bypass time, | 129(109,146) | 123(105,146) | 134(124,143) | 1.01(0.99-1.02) | 0.52 |
| Aortic cross-clamp time, min | 88(71,101) | 88(70,99) | 99(86,101) | 1.02(0.99-1.04) | 0.23 |
| Postoperative |  |  |  |  |  |
| Postoperative IABP implantation | 23(38.3) | 12(26.1) | 11(78.6) | 10.39(2.47-43.68) | 0.001 |
| Postoperative ECMO implantation | 3(5) | 0(0) | 3(21.4) | - | 0.011 |
| Postoperative CRRT implantation | 12(20) | 3(6.5) | 9(64.3) | 25.80(5.20-127.99) | ＜0.001 |
| Re-sternotomy | 3(5) | 1(2.2) | 2(14.3) | 7.50(0.63-89.87) | 0.122 |
| Pulmonary infection | 11(18.3) | 3(6.5) | 8(57.1） | 19.11(3.94-92.60) | ＜0.001 |
| Postoperative ejection fraction  (EF) | 47.4±6.68 | 48.153±5.285 | 44.583±10.211 | 0.92(0.83-1.02) | 0.111 |
| Residual shunt | 5(8.3) | 5(10.9) | 0(0) | - | 0.329 |
